# Early Life History of the ‘Irukandji’ Jellyfish *Carukia barnesi*

**DOI:** 10.1371/journal.pone.0151197

**Published:** 2016-03-08

**Authors:** Robert Courtney, Sally Browning, Jamie Seymour

**Affiliations:** Australian Institute for Tropical Health and Medicine, Division of Tropical Health & Medicine, James Cook University, Cairns, Queensland, Australia; UC Irvine, UNITED STATES

## Abstract

Adult medusae of *Carukia barnesi* were collected near Double Island, North Queensland Australia. From 73 specimens, 8 males and 15 females spawned under laboratory conditions. These gametes were artificially mixed which resulted in fertilized eggs. Post fertilization, most eggs developed to an encapsulated planula stage and then paused for between six days and six months prior to hatching as ciliated planulae. The paused stage planulae were negatively buoyant and adhered to substrate. The first planula was produced six days post fertilization, lacked larval ocelli, remained stationary, or moved very slowly for two days prior to metamorphosis into primary polyps. Mature polyps reproduced through asexual reproduction via lateral budding producing ciliated swimming polyps, which in turn settled and developed into secondary polyps. Medusae production for this species was in the form of monodisc strobilation, which left behind polyps able to continue asexual reproduction.

## Introduction

Cubozoans have a metagenic life cycle which alternates between benthic sessile polyps and motile pelagic medusae [1–8]. It is this alternation of generations that contributes to the often predictable occurrence of cubozoan medusae [4,5,9,10]. There are approximately 50 described species of Cubozoa and of these the early life history of only eight have been described to date [2,3,5–8,10–15]. The polyp stage of cubozoans not only initiates the seasonal onset of medusae, but also allows for population increase through asexual reproduction, which has potential for exponential population growth [5,14,16–18]. Therefore, the success of the polyp stage will drive not only the seasonal timing of medusa but also their abundance.

In northern Australia, cubozoan medusae typically arrive in large numbers associated with increased sea temperatures during the monsoonal months [4,5,9,19,20]. This seasonal cycle in some species has been reported to begin earlier and last longer in areas closer to the equator [9,21,22]. The seasonal timing of one Australian cubomedusae, *Chironex fleckeri*, has been shown to be initiated by metamorphosis of the polyp stage, which presumably uses increasing photoperiod as a cue for metamorphosis [9]. Another highly seasonal Australian cubozoan, both temporally and spatially, is the small carybdeid *Carukia barnesi* [20,23,24]. This species is present in north Queensland waters between November and May, and may be locally abundant or absent for periods of time during the ‘stinger season’ [19,20,23–26].

Little is known about the general ecology and biology of *C*. *barnesi*; however, the medusae stage is considered oceanic, planktonic, has been found around coral reefs or islands, and under certain conditions on beaches [19,20,23,24,26]. A sting from *C*. *barnesi*, as well as several other cubozoans, can cause Irukandji syndrome, which is often severely painful, potentially fatal and may require hospitalization for treatment [19,27,28]. The direct cost in treating envenomed victims is estimated to be between one and three million dollars per year in northern Australia alone, and the negative impact this species has on the Australian tourism industry through reduced revenue is substantial [19]. Understanding the general ecology of the polyp stage of *C*. *barnesi* may allow for the determination of the start of the jellyfish season and elucidate the factors affecting the abundance of medusae present. This could contribute to decreasing the number of envenomed victims per year and reduce the costs associated with treating these stings.

### Aims

The aim of this study was to describe the polyp stage of *C*. *barnesi* from egg fertilization through to medusa detachment and to provide a repeatable method for producing laboratory based polyp cultures of this species for scientific research.

## Method

### Ethics Statement

All specimen collections were conducted in accordance with Permit Numbers: G11/34552.1 and G15/37396.1.

### Specimen Identification

*Carukia barnesi* is a small species in the family Carukiidae with a bell height of up to 35 mm and tentacles up to 1.2 m long [20,24,26,29]. This species has four tentacles, one from each of the four pedalia, a rhopaliar niche with horns, and tentacles that have an alternating pattern of large and small nematocyst crescents that resemble “neckerchiefs” [20,24,26,29]. Little is known about the distribution of this species; however, they are present along the north-eastern coast of Australia from Lizard Island to Fraser Island [20,23,24,26,29].

### Capture Method

Four collection trips were undertaken to capture medusae of *C*. *barnesi* near Double Island, North Queensland, Australia (16°43.5′S, 145°41.0′E) in 2014 and 2015. The collection trips were undertaken on January 4 and 5, 2014, December 15, 2014, and April 7, 2015, between 1900 and 2300hrs, which resulted in the capture of both male and female medusae that spawned approximately nine hours post capture. Three underwater LED lights (7,000 lumens each) were suspended just below the water level from a 5.8 m vessel. As medusae approached the lights they were captured with a net and transferred into individual 500 ml plastic containers, held at ambient temperature, for transport. The sea surface temperature varied from 27.5°C to 30°C with a mean temperature of 29°C. The salinity at the capture location varied between 34‰ to 35‰ and the water depth varied between three to six meters. Post capture, the specimens were transported to the laboratory and held at a constant temperature of 28°C ± 0.5°C in complete darkness.

### Fertilization

After the medusae had been held overnight, a total of 8 females and 15 males spawned from a total of 73 medusae, which was determined by the presence of eggs for females and sperm for males evident within the holding vessels. The spawned medusae were removed from the transport containers that contained the gametes and the water was stirred to suspend the eggs. Approximately one third of the mixture was poured into a three litre container. To this, 100 ml of water that contained male gametes was added. This mixture was stirred briefly and then the three litre container was filled with filtered sea water to reduce the gamete density. One third of this mixture was poured into 15 plastic containers of 70 ml volume. The remaining mixture was then further diluted by approximately 30%, with filtered sea water and poured into 15 additional 70 ml containers. This process was repeated resulting in approximately 250 specimen containers with varying egg densities for each female. The egg densities in the containers ranged from one to 15 eggs per ml. A loose lid was placed on each container to reduce evaporation and the containers were held in darkness at a constant temperature of 28°C.

### Polyp Maintenance

Until the first primary polyps were observed, all containers received a weekly 50% water exchange (taken from the top of the vessels) with filtered sea water and were not fed. Each 70 ml container that contained polyps was fed twice a week with approximately 20 freshly hatched, first instar, decysted *Artemia* nauplii, followed by a 50% water exchange 24 hours later. As the polyp density increased through asexual reproduction, the food density was also increased.

### Culturing Polyps

As the polyp numbers increased through asexual reproduction, freshly budded off swimming stage polyps (similar to those described in *Morbakka virulenta* [6,15]) were harvested. These were extracted by using a pipette to stir the contents of the 70 ml containers prior to each 50% water exchange. The waste water was then poured into a larger container where the swimming polyps could be removed and this was repeated post each feeding event. These swimming polyps were transferred into three 30 litre temperature controlled tanks (28°C). Once the swimming polyps settled and attached, they were fed live *Artemia* nauplii once per week.

### Nematocyst Identification

In order to describe the cnidome of the different developmental stages of *C*. *barnesi*, a series of nematocyst presses were performed, as described below. The sampled stages were: paused stage unhatched planula; hatched planula; primary polyp; mature polyp; swimming stage polyp; and detached one day old medusae. Each sample was placed on a glass microscope slide and a cover slip was then placed over the sample. In most cases this pressure was sufficient to cause nematocyst discharge. In cases where none of the nematocysts discharged, a small quantity of ethanol was added to the sample. Each sample was then viewed on a stereo microscope, photographed and the nematocyst types were identified using the key developed by Rifkin [see 25] then compared to the known nematocyst types present on the adult medusae.

## Results

### Egg Fertilization and Development

**Fig 1:**
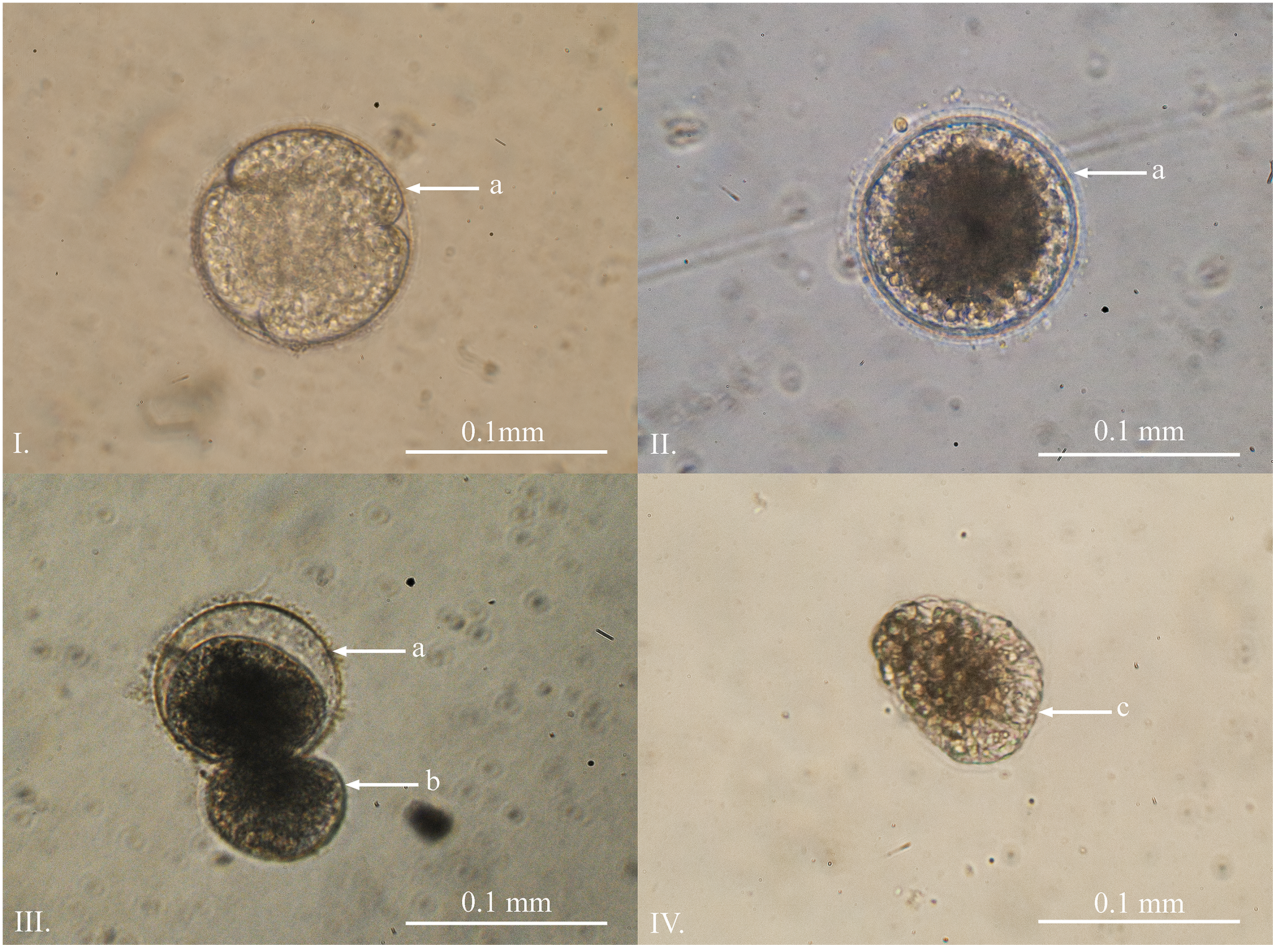
Planula development of *Carukia barnesi*. **I.** Early cell division post fertilisation. **II.** Developed blastula approximately 48 hours post fertilization. **III.** Ciliated planula larvae hatching after a minimum of six days post fertilization. **IV.** Free swimming planula. **Letters indicate:** a) egg capsule; b) emerging planula; c) anterior end.

Unfertilized eggs were translucent and negatively buoyant with a diameter between 0.08 and 0.11 mm (*n* = 10, $\bar{x}$ = 0.089 mm, *SD* = 0.009). Approximately two hours post mixing of the gametes, a fertilization membrane was produced. During initial cell division the eggs remained negatively buoyant, with a diameter between 0.08 and 0.11 mm (*n* = 10, $\bar{x}$ = 0.104 mm, *SD* = 0.010) (Fig 1I). The first blastula was seen approximately 30 hours post fertilization and the majority of the eggs were at the blastula stage within 48 hours. Around 90% of the eggs from each batch were fertilized and reached this stage (Fig 1II). During this time, the eggs/blastulae remained negatively buoyant and stuck to the base of the culture containers. The formation of the planula stage took place within the egg capsules and the planulae could be seen slowly rotating within. The planulae then entered a pausal stage and hatched over a minimum of six days and a maximum time of over six months (Fig 1III). The diameter of the unhatched planulae ranged between 0.094 and 0.120 mm (*n* = 10, $\bar{x}$ = 0.105 mm, *SD* = 0.009).

### Planula

*Carukia barnesi* free swimming planula larvae were first observed six days post fertilization, and lacked larval ocelli. The hatched planulae were heavily ciliated and the beating cilia caused a jittery shaking motion (Fig 1IV). The planulae did not actively swim and appeared stationary, or moved very slowly, during all observations. The planulae were flattened on one end, which was the predominant direction of travel; flat end forward. The length of the planulae ranged from 0.11 to 0.12 mm (*n* = 10, $\bar{x}$ = 0.115 mm, *SD* = 0.005), were negatively buoyant and remained on the base of the containers where they eventually settled and metamorphosed into primary polyps after a minimum of two days post hatching.

### Primary Polyps

**Fig 2:**
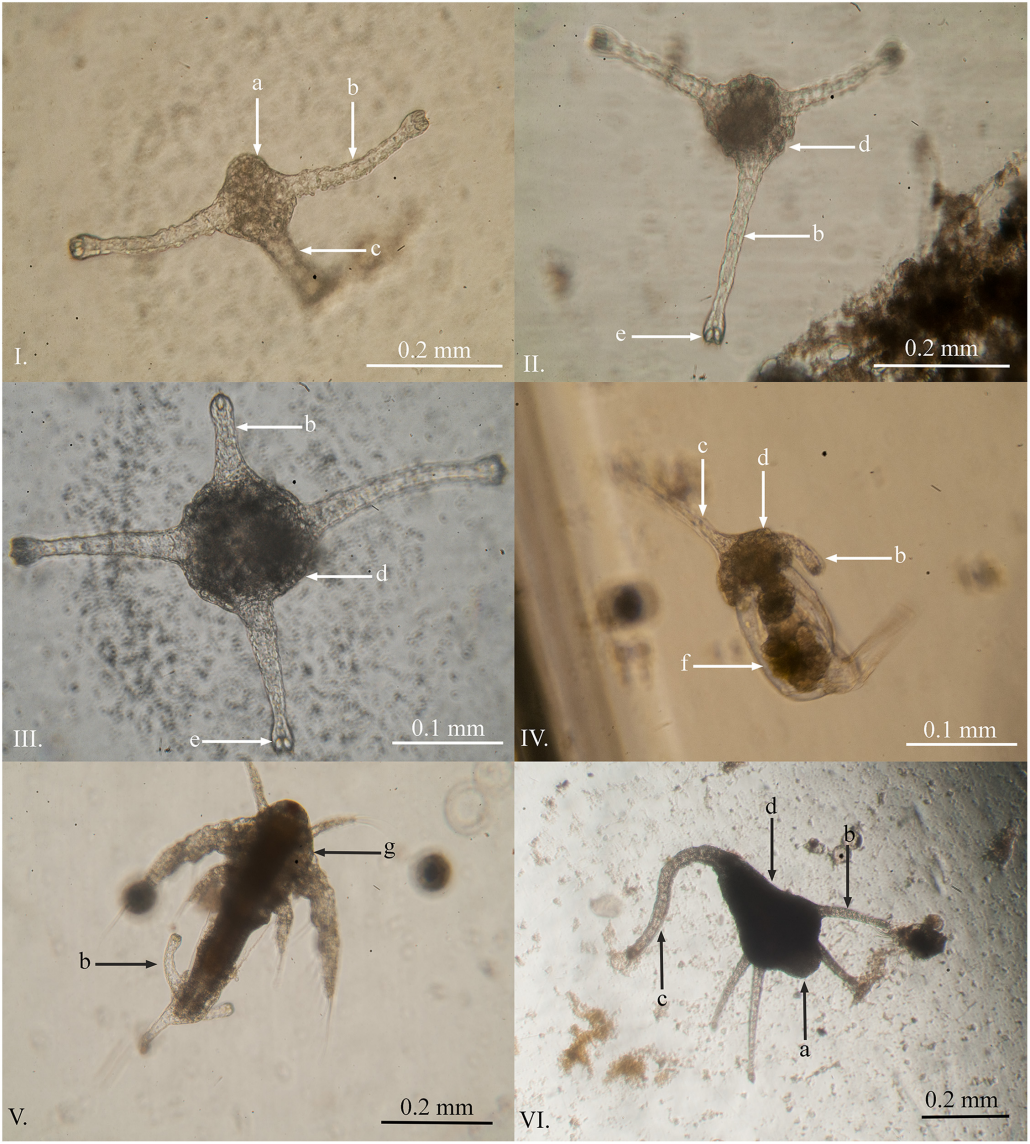
Primary polyps of *Carukia barnesi* at different developmental stages. **I.** Lateral view of a primary polyp at the two tentacle stage. **II.** Vertical view of a three tentacle stage polyp. **III.** Three tentacle stage primary polyp developing a fourth tentacle. **IV.** Three tentacle stage primary polyp feeding on a live rotifer. **V.** Three tentacle stage primary polyp feeding on a live *Artemia* nauplius. **VI.** Four tentacle primary polyp 28 days post fertilization. **Letters indicate:** a) hypostome; b) capitate feeding tentacle; c) stalk region; d) calyx region; e) nematocyst bundle in tentacle tip; f) rotifer; g) *Artemia* nauplius.

Primary polyps of *C*. *barnesi* were first observed eight days post fertilization, two days after the first planula was observed. This species appears to lack a creeping polyp stage and instead develops where the planula settles. First a single tentacle and stalk is formed followed by a second tentacle (Fig 2I). The primary polyps are heavily ciliated and, on occasion, were observed slowly swimming near the base of the containers; however, the majority of polyps remained sedentary. During the first week the primary polyps did not feed even when presented with live or finely chopped *Artemia*, rotifers, fine crayfish meat or boiled chicken egg yolk. Nevertheless, the polyps continued to grow and develop a third (Fig 2II) and fourth tentacle (Fig 2III). At the three tentacle stage the polyps captured live rotifers (Fig 2IV) and/or first instar *Artemia* nauplii (Fig 2V). The primary polyps had the following average dimensions reported in millimetres (*n* = 10): total length ($\bar{x}$ = 0.191, *SD* = .068); stalk length ($\bar{x}$ = 0.113, *SD* = 0.038); calyx length ($\bar{x}$ = 0.078, *SD* = 0.033); calyx width ($\bar{x}$ = 0.075, *SD* = 0.024). The polyps continued to develop until they reached a mature stage (able to asexually reproduce) after approximately 28 days post fertilization (Fig 2VI).

The timeline of these processes is highly variable and only the first observation times have been reported. For example, even within one 70 ml specimen jar after 28 days, it was common to see all of these developmental stages at any given time. Even while there were adult polyps undergoing asexual reproduction, there were still unhatched eggs, hatching planulae, free swimming planulae, and newly formed primary polyps in each container. These eggs continued to hatch within the containers for over six months.

### Polyps and Asexual Reproduction

Mature polyps first underwent asexual reproduction 28 days post fertilization. The basic polyp anatomy consisted of a calyx region that included a motile hypostome which was surrounded by a single circlet of capitate tentacles. There was a distinct demarcation at the junction between the calyx and the stalk region. The stalk region was thin and contractile with a basal disc at the terminal end which anchored the polyp to substrate. All of the external surfaces of the mature polyps were ciliated. The average size of the mature polyps had the following dimensions reported in millimetres (*n* = 10): total length ($\bar{x}$ = 0.885, *SD* = 0.434); stalk length ($\bar{x}$ = 0.560, *SD* = 0.310); calyx length ($\bar{x}$ = 0.325, *SD* = 0.142); calyx width ($\bar{x}$ = 0.254, *SD* = 0.069). The polyps asexually reproduce after having four or more tentacles; however, the number of tentacles on each polyp was highly variable and ranged from four to 24 (*n* = 78, $\bar{x}$ = 11.12, *SD* = 5.31).

**Fig 3:**
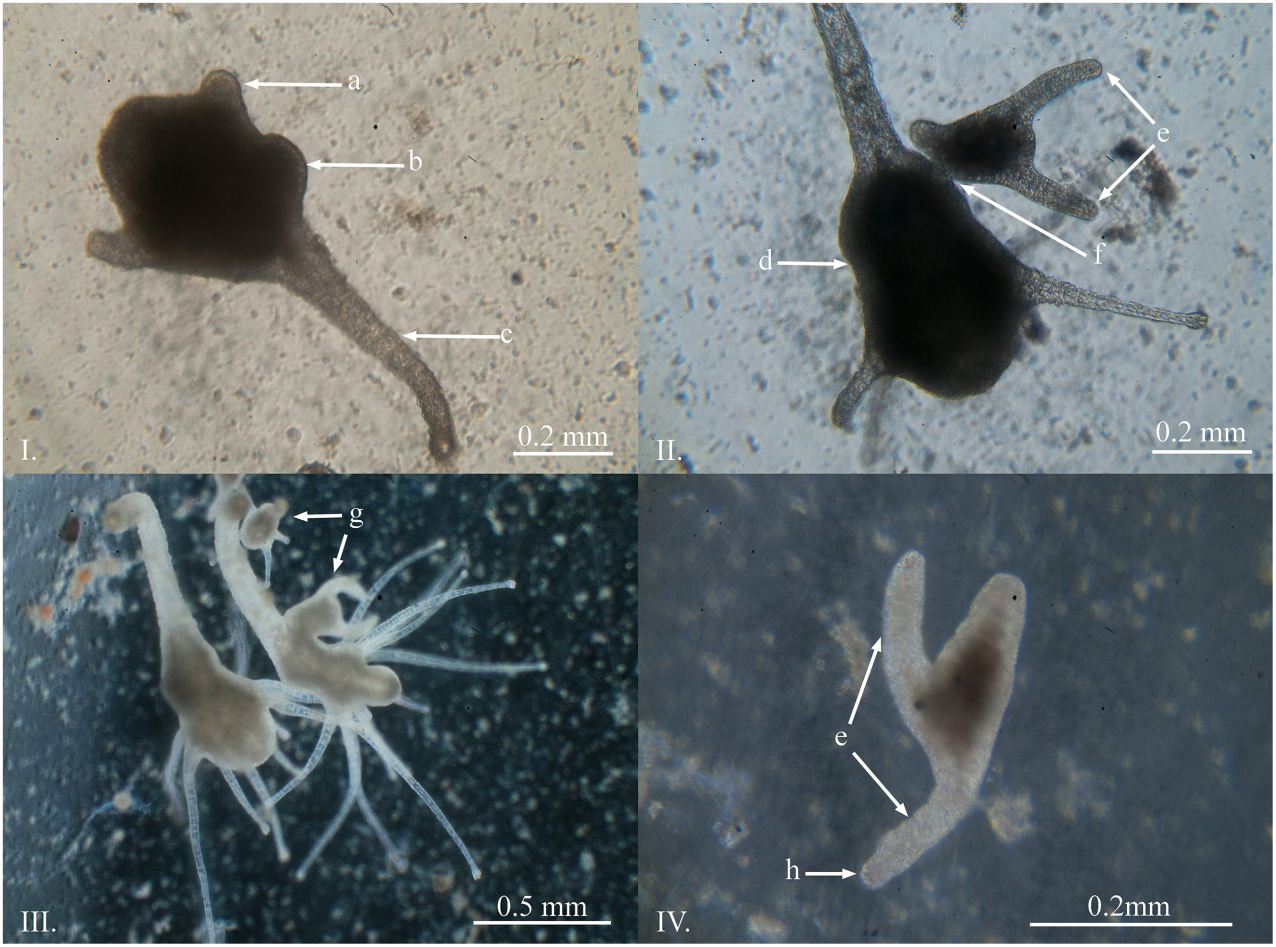
Asexual reproduction of *Carukia barnesi* polyps. **I.** The initial stage of lateral budding. **II.** Formed ciliated swimming stage polyp prior to detachment. **III.** Large mature polyp undergoing asexual reproduction. **IV.** Detached swimming stage polyp. **Letters indicate:** a) retracted tentacle; b) swelling of the calyx where the bud begins to form; c) stalk, d) calyx; e) tentacles of the swimming polyp stage; f) attachment point; g) buds produced through asexual reproduction, h) location of nematocysts in the tentacle tips.

The primary mode of asexual reproduction observed was through the lateral budding of a ciliated swimming polyp similar to those produced by *M*. *virulenta* [6,15]. This process was first evident by a round protrusion originating from the side of the calyx (Fig 3I). The bud then develops two tentacles and a stalk and remains attached to the parent polyp along the calyx (Fig 3II and 3III). This process took approximately four days from the beginning of the bud through to detachment of the free swimming secondary polyp (Fig 3IV). Frequently, polyps were observed producing multiple buds simultaneously, up to five at a time.

The swimming polyps were highly variable in size and ranged from 0.17 to 0.50 mm (*n* = 10, $\bar{x}$ = 0.298 mm, *SD* = 0.106), measured along the latitudinal axis from between the tentacles to the terminal end of the stalk, where small polyps produced small swimming polyps and larger polyps produced larger swimming polyps. The polyps swam along the bottom of the containers, and also up the sides, with one tentacle forward in the direction of travel. The swimming polyps moved like this for one to seven days prior to settlement.

### Medusa Production

Medusa production has only been observed three times to date and only from the first polyp culture, which was fertilized in January 2014. The quantities and dates of medusa production were; 24^th^ of June 2014 (one medusa, 170 days post fertilization), 26^th^ of August 2014 (approximately 100 medusae, 233 days post fertilization) and 20^th^ of May 2015 (approximately 50, medusae 500 days post fertilization). The polyp culture, both the original cultures and the three 30 l temperature controlled tanks, included more than one million polyps (determined by extrapolation), and due to this, the actual percentage of polyps that underwent medusa production on these dates was minimal.

**Fig 4:**
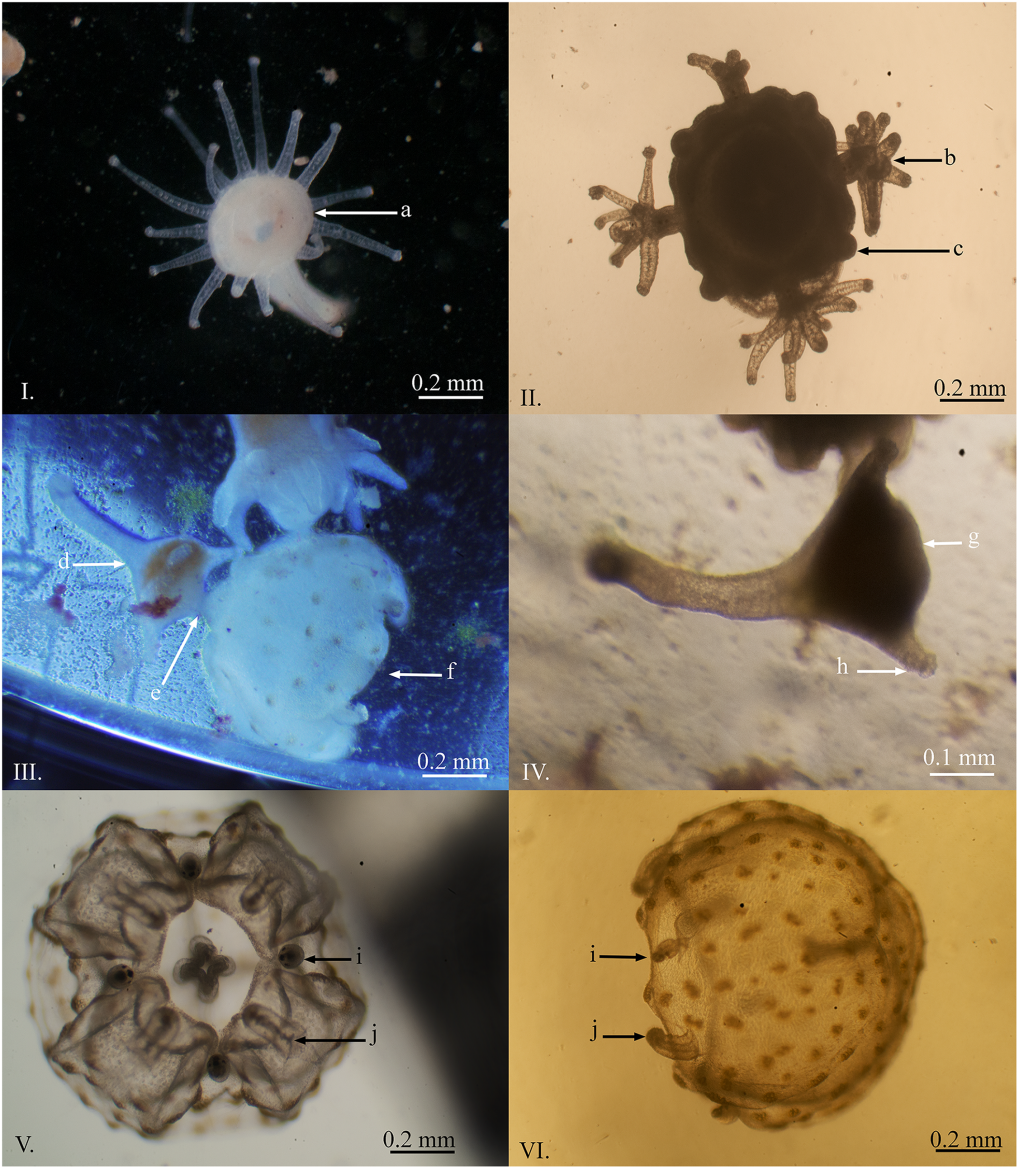
Medusae production of *Carukia barnesi*. **I.** Initial stage of medusae production, note the change in shape of the hypostome region. **II.** The tentacles migrate to four opposing sections of the forming bell, fuse, and begin to form the rhopalia. **III.** The bell begins to pulse and a narrowing divides the bell of the forming medusae from the hypostome of the original polyp. **IV.** The remaining polyp after completed medusae production. **V.** Oral view of a newly detached medusa. **VI.** Lateral view of a newly detached medusa with pigmented nematocyst batteries on the bell. **Letters indicate:** a) change in shape of the hypostome region; b) fusing of tentacles which begin to form rhopalia; c) early development of medusa tentacles; d) polyp producing a medusa through monodisc strobilation; e) connective tissue between the polyp and forming medusa; f) pulsing medusa approximately 24 hours prior to detachment; g) hypostome; h) feeding tentacle; i) formed rhopalia; j) short feeding tentacles.

Medusa production in *C*. *barnesi* occurred in the form of monodisc strobilation similar to that observed in *M*. *virulenta* described by Toshino et al. [15]. First, the stalk region contracted and the oral disc widened, the calyx also began to extend (Fig 4I). The tentacles migrated to four equally spaced corners of the forming bell. The tentacles then fused together at the base and a dark pigmentation of the forming rhopalia was visible (Fig 4II). The tentacles were further reabsorbed and the rhopalia continued development. At this time, the calyx became constricted and tentacles began to develop below the developing medusa. The manubrium then formed, nematocyst warts became visible on the forming bell, small pedalia formed, and there was a further separation between the forming medusa and the original polyp (Fig 4III). The medusa began to pulse approximately two days prior to detachment.

The detached medusae were positively phototactic, congregated on the surface of the containers, and had a bell height that ranged between 0.33 to 0.80 mm (*n* = 8, $\bar{x}$ = 0.560 mm, *SD* = 0.140), and a bell width that ranged between 0.45 to 0.83 mm (*n* = 10, $\bar{x}$ = 0.568 mm, *SD* = 0.110) (Fig 4V and 4VI). The nematocyst warts were clearly visible on the external surface of the bell. The rhopalia were developed prior to detachment, and the beginnings of small feeding tentacles were visible. The polyps that were left behind, after strobilation, had between four and eight tentacles (Fig 4IV). These polyps continued to grow and returned to production of swimming polyps. No polyps were observed producing multiple medusae at one time. The medusae were able to consume *Artemia* nauplii soon after detachment; however, survival was limited (five days).

### Nematocysts of Polyps and Early Stage Medusae

There were two different types of nematocysts found in the early developmental and polyp stages which consisted primarily of homotrichous microbasic tumiteles primarily located within the tentacle tips of the polyps and spherical isorhizas in the body of the polyps. These are the same nematocyst types identified in the medusae stage of this species, which consists primarily of homotrichous microbasic tumiteles on the tentacle nematocyst batteries and spherical isorhizas on the bell nematocyst batteries [24–26]. The cnidome of the unhatched and hatched planulae consisted of only homotrichous microbasic tumiteles, whereas both nematocyst types were present in primary polyps, mature polyps, swimming polyps, and newly detached medusae. The sizes of each nematocyst type, at each life stage, are as follows (with measurements recorded in microns): hatching planula tumiteles (*n* = 5, 3.1 to 4.0, $\bar{x}$ = 3.6, *SD* = 0.37); primary polyp tumiteles (*n* = 7, 11.9 to 13.2, $\bar{x}$ = 12.6, *SD* = 0.50); primary polyp isorhizas (*n* = 7, 4.0 to 6.2, $\bar{x}$ = 4.6, *SD* = 0.80); mature polyp tumiteles (*n* = 10, 10.1 to 12.8, $\bar{x}$ = 11.2, *SD* = 0.89); mature polyp isorhizas (*n* = 7, 4.0 to 5.3, $\bar{x}$ = 4.7, *SD* = 0.61); swimming polyp tumiteles (*n* = 8, 11.0 to 11.9, $\bar{x}$ = 11.3, *SD* = 0.31); swimming polyp isorhizas (*n* = 5, 6.6 to 7.9, $\bar{x}$ = 7.0, *SD* = 0.57); freshly detached medusa tumiteles (*n* = 10, 11.0 to 14.1, $\bar{x}$ = 12.1, *SD* = 1.00); freshly detached medusa isorhizas (*n* = 10, 4.8 to 5.3, $\bar{x}$ = 5.1, *SD* = 0.23).

## Discussion

On four occasions the adult medusae of *C*. *barnesi* collected from Double Island spawned approximately nine hours post capture while held at a constant temperature of 28°C in darkness in water collected from the sample site. Although all attempts were made to replicate the conditions at the collection site, it is expected that this was a stress induced spawning event and these specimens may not have spawned *in-situ* over the same timeframe. Although, *C*. *barnesi* are routinely collected during the summer months at this location, specimens have never been observed spawning at this, or any other location, nor has it been reported in the literature. This is unlike the known predictable spawning aggregations of *Alatina alata* that occur 8 to 10 days after a full moon in Hawaii [11,30–33]. Furthermore, the eggs of some cubozoan species are fertilized internally [7,8,12], and because the fertilization method implemented in this study was artificial, the possibility that there is more to the reproductive behaviour of *C*. *barnesi* should not be discounted.

The size and developmental timing of the fertilized egg phase through to gastrulation was similar to other cubozoans [5–7,11,12,15]. The first notable difference was the dormant phase of the unhatched *C*. *barnesi* planulae. After the planula developed within the egg capsule, it entered a dormant phase that lasted six days to over six months. During this time the unhatched planulae were negatively buoyant and stuck to the base of the containers. A similar dormant pause stage has been described in *M*. *virulenta*, where encysted blastulae lasted seven to 21 days before hatching as either free swimming planulae, or as primary polyps [6,15]. The paused stage planulae of *C*. *barnesi* only hatched as free swimming planulae. The function of the encapsulated pause stage is unknown; however, it may stagger the hatching time, potentially reducing the impact of intraspecific competition. There is also the possibility that there is an environmental cue, such as a change in temperature, photoperiod, or substrate, which triggers synchronous hatching.

The hatched planulae of *C*. *barnesi* were negatively buoyant, lacked larval ocelli, moved very slowly on the base of the containers and had nematocysts. Cubozoan planula have previously been reported as being negatively buoyant [5–7,11,12,15], either possessing larval ocelli [6,11,34,35], or lacking larval ocelli [6,15], and having nematocysts which were used for attachment to substrate [5]. This use of nematocysts in the planula of *C*. *barnesi* was not observed and the function of the nematocysts during the planula stage is unknown, but is presumably for defence. The lack of larval ocelli and the limited swimming capacity of *C*. *barnesi* planulae may only provide limited dispersal potential that does not require photoreceptors for orientation or settlement choice.

The primary polyps of *C*. *barnesi* were able to develop to the four tentacle stage without food. This suggests that the eggs either contain sufficient stores to reach this stage, or the planulae and/or primary polyps may be able to uptake dissolved organic material directly from the water column. Once the primary polyps began to feed on *Artemia* nauplii they rapidly developed into mature polyps which began asexual reproduction through lateral budding. This process was very similar to the asexual reproduction method seen in *M*. *virulenta* from Japan, which produced small swimming stage polyps [6,15]. The swimming stage polyps of *C*. *barnesi* were ciliated and swam along the base and up the vertical surfaces of the culture containers prior to settlement and transformation into a secondary polyp. The mobility of the swimming stage polyps may not only provide a dispersal mechanism, but may also allow for increased selectivity in settlement position and/or substrate choice. Also, the size of the swimming polyps was highly variable, where larger polyps produced large swimming polyps and smaller polyps produced small swimming polyps.

The process of medusa production in *C*. *barnesi* was also very similar to the monodisc strobilation recently discovered in *M*. *virulenta* [15]. Medusa production of cubozoans is highly variable between species ranging from complete metamorphosis of the polyp into a medusa [2,3,8,10,11,14,36], leaving behind a small amount of regenerative material (residuum) [37], and monodisc strobilation which may leave behind a developed polyp (with tentacles) able to continue asexual reproduction [15]. This is a reproductive event, which may suggest that the polyps of *C*. *barnesi* may reside in a location that allows for the annual persistence of the polyp.

The cnidome of the early life stages of *C*. *barnesi* was found to be identical to the cnidome of the adults. This deviates from other cubozoan species which change their cnidome between polyp and medusa stages [11,14].

Cubozoans have been shown to be sophisticated in many areas of their ecology from possessing complex vision capabilities [38–40], prey capture techniques [41], behavioural patterns [42,43], and complex venoms [19,28,44]. Not surprisingly, the life cycle of cubozoans also have many species specific complexities [2,3,6,7,10,13–15]. In order to investigate cubozoan life cycles, production of lab-based polyp cultures is essential. From these cultures the effects of varying parameters such as temperature, salinity, and feeding frequency can be explored to elucidate the most likely habitats that support polyp proliferation. This could then be used to determine where the polyp colonies may reside *in-situ*. These parameters could also be experimentally pursued to elucidate cues for medusae production which may give better insight into what factors drive the strong seasonal occurrence of this, and other, cubozoan species.
